# Progesterone mediates brain functional connectivity changes during the menstrual cycle—a pilot resting state MRI study

**DOI:** 10.3389/fnins.2015.00044

**Published:** 2015-02-23

**Authors:** Katrin Arélin, Karsten Mueller, Claudia Barth, Paraskevi V. Rekkas, Jürgen Kratzsch, Inga Burmann, Arno Villringer, Julia Sacher

**Affiliations:** ^1^Department of Neurology, Max Planck Institute for Human Cognitive and Brain SciencesLeipzig, Germany; ^2^Clinic of Cognitive Neurology, University of LeipzigLeipzig, Germany; ^3^Leipzig Research Center for Civilization Diseases, University of LeipzigLeipzig, Germany; ^4^Centre for Addiction and Mental Health Research Imaging Centre and Campbell Family Mental Health Research Institute at the Centre for Addiction and Mental Health and the Department of Psychiatry, University of TorontoToronto, ON, Canada; ^5^Clinical Chemistry and Molecular Diagnostics, Institute for Laboratory Medicine, University Hospital LeipzigLeipzig, Germany; ^6^Integrated Research and Treatment Center Adiposity Diseases, University of LeipzigLeipzig, Germany; ^7^Berlin School of Mind and Brain, Mind and Brain Institute, Humboldt University BerlinBerlin, Germany

**Keywords:** menstrual cycle, RS-fMRI, functional connectivity, estradiol, progesterone

## Abstract

The growing interest in intrinsic brain organization has sparked various innovative approaches to generating comprehensive connectivity-based maps of the human brain. Prior reports point to a sexual dimorphism of the structural and functional human connectome. However, it is uncertain whether subtle changes in sex hormones, as occur during the monthly menstrual cycle, substantially impact the functional architecture of the female brain. Here, we performed eigenvector centrality (EC) mapping in 32 longitudinal resting state fMRI scans of a single healthy subject without oral contraceptive use, across four menstrual cycles, and assessed estrogen and progesterone levels. To investigate associations between cycle-dependent hormones and brain connectivity, we performed correlation analyses between the EC maps and the respective hormone levels. On the whole brain level, we found a significant positive correlation between progesterone and EC in the bilateral dorsolateral prefrontal cortex (DLPFC) and bilateral sensorimotor cortex. In a secondary region-of-interest analysis, we detected a progesterone-modulated increase in functional connectivity of both bilateral DLPFC and bilateral sensorimotor cortex with the hippocampus. Our results suggest that the menstrual cycle substantially impacts intrinsic functional connectivity, particularly in brain areas associated with contextual memory-regulation, such as the hippocampus. These findings are the first to link the subtle hormonal fluctuations that occur during the menstrual cycle, to significant changes in regional functional connectivity in the hippocampus in a longitudinal design, given the limitation of data acquisition in a single subject. Our study demonstrates the feasibility of such a longitudinal Resting-state functional Magnetic Resonance Imaging (rs-fMRI) design and illustrates a means of creating a personalized map of the human brain by integrating potential mediators of brain states, such as menstrual cycle phase.

## Introduction

Sex hormones influence both brain and behavior and are potent modulators of brain plasticity across the life-span (Peper et al., 2011; Galea et al., 2013). Several lines of evidence from animals and humans suggest sex hormones stimulate neurogenic cascade processes by promoting neurite outgrowth (Minano et al., 2008), mitochondrial and synaptic health (Hara et al., 2014), dendritic branching (Hao et al., 2006) and myelination (Patel et al., 2013), thus playing a pivotal role in structural brain organization. Neuroimaging studies in humans have begun to address the question of whether the effect of sex hormones on neural plasticity is reflected in structural connectivity changes. Supporting evidence stems from reports of increased hippocampal volume in postmenopausal estrogen therapy users (Eberling et al., 2003; Boccardi et al., 2006; Lord et al., 2008), as well as from studies demonstrating hormonal contraceptive use to be associated with larger gray matter volumes in cortical regions (Pletzer et al., 2010; De Bondt et al., 2013a), and with changes in cerebral white matter (De Bondt et al., 2013b).

The menstrual cycle offers a unique experimental-setup for studying the potential effects of subtle physiological fluctuations of sex hormones on structural or functional brain architecture. FDG-glucose positron emission tomography (PET) data suggests that, in primates, fluctuations of ovarian hormones across the menstrual cycle influence activity in brain areas involved in the processing and regulation of emotion (Rilling et al., 2008). Glucose metabolism in the human brain also displays changes associated with the menstrual-cycle (Reiman et al., 1996). Reiman and colleagues found significantly higher glucose metabolism in the thalamus, as well as the prefrontal, temporo-parietal, and inferior temporal cortex during the mid-follicular phase, when estrogen levels are high. In contrast, higher metabolic rates were found in the anterior insula, as well as the superior temporal, anterior temporal, occipital, cerebellar, and cingulate cortex, during the mid-luteal phase (Reiman et al., 1996). A study using PET with high-specific-activity [^11^C]raclopride showed sex differences in dopamine release between men and women (Munro et al., 2006). Baseline striatal dopamine binding potential and dopamine release in men and women following amphetamine and placebo challenges were compared. There was no sex difference in baseline binding potential, but men had greater dopamine release than women in the ventral striatum and additionally in three of four striatal regions examined. Differences were revealed in the anterior putamen, as well as the anterior and posterior caudate nuclei, but not in the posterior putamen (Munro et al., 2006).

In female cynomolgus monkeys an effect of menstrual cycle phase on dopamine D2 receptor availability in the caudate nucleus and putamen could be demonstrated (Czoty et al., 2009). Using the selective D2-like receptor ligand [^18^F]fluoroclebopride PET scans were conducted during the luteal phase and during the follicular phase of the menstrual cycle. Distribution volume ratios for the caudate nucleus and putamen were calculated using the cerebellum as the reference region and were used as a measure of D2 receptor availability. [^18^F]fluoroclebopride distribution volume ratios were significantly higher in the luteal phase compared to the follicular phase in both the caudate nucleus (11.7% difference, *p* = 0.02) and putamen (11.6% difference, *p* = 0.03) (Czoty et al., 2009). The authors conclude that menstrual cycle may influence striatal dopamine receptor availability.

Resting-state functional Magnetic Resonance Imaging (rs-fMRI) focuses on the assessment of spontaneous low frequency fluctuations in brain activity, in the absence of a task (Biswal et al., 1995). Measures of connectivity between these spontaneous fluctuations have been shown to reflect communication across large-scale networks in the human brain (Nir et al., 2008; Biswal et al., 2010; Keller et al., 2013). Further, sexual dimorphism has been described for these intrinsic connectivity patterns (Biswal et al., 2010; Tian et al., 2011). Given the wide expression of receptors for both estrogen and progesterone in the human brain, including many highly interconnected regions (McEwen, 2002; Brinton et al., 2008; Weiser et al., 2008), fluctuations of ovarian hormones related to the menstrual cycle likely influence the nature of communication between these brain regions. Intrinsic functional connectivity based on fMRI is especially sensitive to such coupling dynamics and can provide information about network relationships on a whole brain level (Buckner et al., 2013).

One common method to assess global network connectivity is to calculate eigenvector centrality (EC) (Lohmann et al., 2010; Sato et al., 2014), a graph-based measure of centrality that also takes the centrality of nodes that it connects to into account (Bonacich, 1972). In graph theory, a network is defined as a collection of items (nodes) that possesses pairway relationships (edges) (Sato et al., 2014). The EC of a node is proportional to the EC of the nodes in its neighbors and measures how the neighbors of a node are connected to the network (Bonacich, 1972). Moreover, EC quantifies the hierarchical relevance of a node. As we aim for a whole brain investigation of hierarchical network changes across the menstrual cycle, we chose not to apply other graph-based centrality methods such as degree, closeness or betweeness, which are less suitable for whole brain maps due to computational complexity (Lohmann et al., 2010). Furthermore, we refrain from using model-free approaches, such as independent component analysis (ICA), as for successful ICA-application substantial a posteriori selection of valid components must be made (reviewed by Margulies et al., 2010). Instead, EC is parameter-free, computationally fast and does not depend on prior assumptions (Lohmann et al., 2010). In a functional context, EC has been shown to be more sensitive to paralimbic and subcortical regions (brain regions particularly rich in sex hormone receptor density) (Zuo et al., 2012). Changes in EC signal have previously been linked to developmental changes of the brain (Sato et al., 2014), changes in motor function (Taubert et al., 2011) and pharmacologically induced changes in neurotransmitter levels (Schaefer et al., 2014). We acknowledge that EC is a measure that can be viewed as difficult to relate to function in a particular cognitive domain or to specific behavioral aspects. Buckner et al. (2013) propose in their expert-consensus statement that intrinsic functional connectivity provides a powerful and unique tool to provide insight into human brain organization. They further discuss this technique as based on an inherently ambiguous measure that reflects constraints both from static anatomical connectivity and from poorly understood dynamic functional coupling changes and recommend using this measure as a suitable tool for generating hypotheses about brain organization that will require further study.

To date, two recent studies have investigated resting state connectivity data across the menstrual cycle in humans (Petersen et al., 2014; Hjelmervik et al., 2014). The first (Hjelmervik et al., 2014) did not find any evidence for functional connectivity changes across the menstrual cycle. However, the authors limited the monitoring of the menstrual cycle to subjective subject-reporting, assessment of sex hormone fluctuation across the menstrual cycle to the collection of saliva samples of estrogen and progesterone at three time-points and did not include any LH measurements to further confirm ovulation. The analysis of the second study (Petersen et al., 2014) was constrained to the anterior default mode network (aDMN) and the executive control network, in a between-group design. The authors found greater connectivity between the aDMN and the angular gyrus in women scanned in the follicular phase vs. women scanned in the luteal phase (Petersen et al., 2014).

There are also EEG-measurements in resting humans (rest is typically dominated by alpha oscillations): Brotzner et al. (2014) correlated frequency of alpha oscillations in resting women with menstrual cycle phase, sex hormone level, or use of oral contraceptives and suggest a modulation of resting alpha frequency by endogenous estradiol. There was no association with endogenous progesterone in this study. Luteal women showed highest and late follicular women showed lowest individual alpha frequency or center frequency that both correlated negatively with endogenous estradiol level, but not with endogenous progesterone (Brotzner et al., 2014).

Several important conclusions on the role of sex hormones in neuroplastic adaptation have also been drawn from studying the menstrual cycle in animal models, such as rats (Tanapat et al., 1999; Scharfman et al., 2003) or macaque monkeys (Czoty et al., 2009). However, in contrast with the human cycle, the rat cycle is much shorter, consisting of 4–5 days, and also the macaque estrus displays distinct differences compared to the human cycle that can complicate direct translation of such findings across species. Thus, there is substantial need for detailed studies *in vivo* in the human menstrual cycle. Most studies that demonstrate menstrual cycle related changes in brain structure and function report such observations in the context of another main hypothesis aimed at comparing men or women or women on and off oral contraception (Pletzer et al., 2010; De Bondt et al., 2013b; Petersen et al., 2014).

One of the reasons explaining this lack of neuroimaging data on the impact of the menstrual cycle on the human brain is likely tied to the challenges of thoroughly monitoring the menstrual cycle. An extensive body of literature from research on accuracy for reports of the last menstrual period before a pregnancy suggests that subjective menstrual diaries are highly prone to inaccuracies. For example, nearly 50% of women enrolling in a study examining routine ultrasound screening for prediction of gestational age had suspect menstrual histories (Campbell et al., 1985). Only 39% of women correctly reported their last menstrual bleeding when the time of recall was 3 weeks or longer, most women tended to underreport the length of their menstrual cycles at that time (Wegienka and Baird, 2005). Daily measurements of core body temperature have been proposed as a non-invasive method to monitor the menstrual cycle. However, this method has also been demonstrated to be prone to error and misreports of cycle-length because of the day-to-day variability of temperature readings, cycle variability and the effects of illness, medication, diet and changes in sleeping patterns (Bauman, 1981; Leader et al., 1985). The menstrual cycle can be divided into two main phases: the follicular phase between onset of menses and ovulation with rising levels of estrogen and very low levels of progesterone, and the luteal phase, starting after ovulation until the onset of the next menses characterized by high levels of progesterone and low levels of estrogen, especially premenstrual. Thus, detection of sex hormone concentrations in blood at single time-points to differentiate the follicular from the luteal phase is not a feasible solution, either, as very similar estrogen concentrations can be found during the late follicular and the mid-luteal phase and more frequent samples are needed to establish luteal-phase length and adequacy. Confirmation of luteinizing hormone (LH)-concentration peak in urine samples collected at mid-cycle (12–16 days in a typical cycle) represents a relatively non-invasive option to determine ovulation, because an LH surge is a necessary prerequisite for ovulation. To accurately stage the menstrual cycle phase for the current project we used a combination of all of these methods.

Detailed longitudinal studies of individual cases with rs-fMRI are not common, however there is evidence supporting such a longitudinal approach to be useful. One famous example derived from the field of structural imaging is the single-subject template “Colin's Brain” (Holmes et al., 1998), a widely used brain atlas based on 27 longitudinal scans of the same individual that allows for a high resolution and fine structural details to be seen.

Our current research was motivated by the lack of data on the potential impact of the menstrual cycle on functional brain architecture as a primary research question. To our knowledge no study to date has addressed the question whether menstrual cycle related changes in endogenous sex hormones impact intrinsic connectivity in the female brain using an extensive intra-individual longitudinal design on a whole brain level. The purpose of applying such a study-design is to address the following aspects: (1) The menstrual cycle offers a unique experimental set-up to study changes in endogenous sex hormones. However, these changes are expected to be subtle, thus an intra-individual longitudinal design will be beneficial to adequately capture such potential effects. (2) The menstrual-related changes are expected to occur within hours or days and recent evidence points toward feasibility to visualize changes in intrinsic connectivity following alteration in the neurochemistry of the brain on such a short-time scale (Schaefer et al., 2014). The implementation of shortly-timed intervals of scanning-sessions is a pre-requisite to assess changes on such a short time-scale. We timed our scanning intervals to occur every 2–3 days to account for this requirement.

The aim of the current study was to test the feasibility of such an intra-individual longitudinal design by calculating EC, a measure of intrinsic connectivity across the whole brain. We hypothesize the physiological fluctuations of endogenous estrogen and progesterone levels during the menstrual cycle to significantly impact intrinsic connectivity across the entire female brain.

## Methods

We repeatedly scanned a single, healthy 32-year old female subject with a documented history of regular menstrual cycles. Exclusion criteria were a history of psychiatric and neurological illness or the suffering from other chronic illnesses. The participant was screened using the Structured clinical interview for DSM to disqualify any Axis I major mental disorders (First et al., 1955a), supplemented by the exclusion of Axis II personality disorders (First et al., 1995b). Additionally, we used the Hamilton Rating Scale for Depression (HAM-D) to detect symptoms of clinical depression (Hamilton, 1960), the Mood Spectrum self-report (MOODS-SR) to exclude any subclinical manic-hypomanic symptoms (Dell'Osso et al., 2002), and the State-Trait anxiety inventory (STAI) to screen for clinical anxiety-symptoms (Spielberger and Vagg, 1984). The subject was at a normal weight (BMI = 20.2), antidepressant-naive, free of any medication including any contraceptives, and never pregnant or breast-feeding. Furthermore, the participant was a non-smoker and did not have any current or history of alcohol or any other substance abuse. The participant provided written informed consent to participate. Study and recruitment procedures were carried out in accordance with the Declaration of Helsinki and approved by the research ethics board of the University of Leipzig (EK-No.: 246—2009—09112009).

Following the MRI acquisition at each scan session, fasting blood samples were taken to determine serum hormone levels of estrogen, progesterone, LH, and cortisol. Blood samples were analyzed at the Institute for Laboratory Medicine of the University Hospital Leipzig by the fully automated Roche Cobas® system (Roche, Basel, Switzerland). All samples were measured within one plate; intra-assay coefficients of variance (CV) were within 3.2–6% for estrogen, 2.3–5.2% for progesterone, 1.6–2% for LH and 2.1–5.9% for cortisol. Menstrual cycles were further monitored by LH surge ovulation tests (Diagnostik Nord GmbH hLH-K20 hLH Kassettentest).

In order to detect any potential influence that mood or stress levels may have on resting state connectivity, the following scales were administered prior to every scan: the Visual Analog Scale (VAS) (Grant et al., 1999), the Profile of Mood states (PoMS) (Pollock et al., 1979), the Perceived Stress Scale (PSS) (Cohen et al., 1983), and a standardized assessment of implicit positive and negative affect (IPANAT) (Quirin et al., 2009).

## MRI data acquisition

Imaging was performed each second or third day across four menstrual cycles: each session was started at a different menstrual cycle day to control for any potential scanner-drift artifacts. Thus, each scanning session provides data for one full menstrual cycle scanned across two menstrual cycles (session 1: January 2012, session 2: November 2012), resulting in 16 scans per session and a total of 32 longitudinal scans of the same individual. To control for circadian rhythm effects, every scan was collected at the same time of the day (7:30 am).

All 32 MRI sessions were performed on a 3-Tesla Magnetom Verio scanner (Siemens, Erlangen, Germany) equipped with a 32-channel head array coil. In each session, resting-state fMRI data were acquired using a gradient-echo echo-planar imaging (EPI) sequence. The subject was asked to stay awake, relax, and look at a low-contrast fixation cross. The following parameters were used: 300 whole brain volumes, acquisition matrix = 64 × 64, slice thickness = 4 mm (0.8 mm gap), resulting in a nominal voxel size of 3 × 3 × 4.8 mm^3^. Further imaging parameters: 30 axial slices, TR = 2000 ms, TE = 30 ms, flip angle = 90° and bandwidth = 1954 Hz/pixel. The total scanning time for resting-state fMRI was 10 min.

In addition, for the purpose of image registration and normalization, high-resolution T1-weighted images were acquired using a three-dimensional Magnetization-Prepared Rapid Gradient Echo (MPRAGE) sequence. The Alzheimer's Disease Neuroimaging Initiative (ADNI) standard protocol was used with the following parameters: TI 900 ms, TR 2300 ms, TE 2.98 ms, flip angle 9°, band width 238 Hz/pixel, image matrix 256 × 240, 176 partitions, FOV 256 × 240 × 176 mm^3^, sagittal orientation, average voxel size 1 × 1 × 1 mm^3^.

## Data analysis

Preprocessing of all 32 resting-state fMRI data sets was performed using SPM8 (Wellcome Department of Imaging Neuroscience, London, UK) rev. 8.4010 using (Matlab™ 7.11) including estimation and correction for motion and EPI deformation. Functional images were then co-registered with the high-resolution anatomical image, and normalization was performed using the unified segmentation approach (Ashburner and Friston, 1997). Following normalization, the resulting voxel size of the functional images was interpolated to an isotropic voxel size of 3 × 3 × 3 mm^3^. In the final step of the preprocessing, the functional images were spatially smoothed with a Gaussian kernel of 8 mm full width at half maximum (FWHM).

To identify cycle-dependent connectivity changes, EC mapping (Lohmann et al., 2010) was performed using the LIPSIA software package (Lohmann et al., 2001). EC is able to detect central hubs within a brain network using an algorithm similar to Google's PageRank algorithm, which highlights websites most often linked to other highly interconnected websites (Brin and Page, 1998). Similarly, we were interested in brain regions of high individual connectivity that are, in addition, strongly connected to other regions of high connectivity (i.e., functional brain-hubs). Using EC mapping, we investigated potential correlations between cycle-dependent sex hormone changes and intrinsic functional connectivity. To restrict the EC analysis to meaningful gray matter regions, a mask was generated using the gray matter segmentation of the subject's high-resolution T1-weighted image. This gray matter segmentation was smoothed with 4 mm FWHM, and voxels showing a minimum gray matter probability of 0.12 were included in subsequent EC analysis. For all voxels within this mask, a similarity matrix was generated including Pearson's correlation coefficient between all resting-state fMRI time courses. In order to use a similarity matrix with only positive numbers, a value of 1 was added to all matrix entries before computing the EC. According to the theorem of Perron and Frobenius, this similarity matrix has a unique real largest eigenvalue, and the corresponding eigenvector has strictly positive components (Perron, 1907). Then the EC map was generated using the components of this eigenvector to determine the EC of all voxels.

For each resting-state measurement, EC analysis was computed separately resulting in a set of 32 EC maps. Statistical analysis was performed with SPM8 based on the general linear model using a factorial design. The design matrix was build using columns coding both scanning periods and further covariates including ovarian hormone and cortisol levels. A contrast was specified in order to test for positive or negative correlations between hormones and EC. The resulting statistical parametric maps were processed using a voxel-wise threshold of *p* < 0.001, and remaining clusters were shown with correction for multiple comparisons using family-wise error (FWE) correction at *p* < 0.05. FWE (family wise error) correction for multiple comparisons was applied correcting for *n* = 575 (the number of resells = resolution elements that remain after smoothing the image of the whole brain that contained 61940 voxels in total).

EC mapping is a measure of the interconnectedness of a brain region with all other nodes. As it does not explain whether connectivity changes are specific to another brain region, or reflect a regionally unspecific and global change in connectivity, we performed seed-based correlation analyses. To this end, we placed seed-regions in the left and right DLPFC, and in the left and right sensorimotor cortex. We fed the resulting correlation maps into a statistical analysis using the general linear model and included progesterone level as a covariate in the design matrix. A contrast was specified in order to test for a positive correlation between progesterone levels and connectivity changes obtained by the seed-based correlation analyses. The resulting statistical parametric maps were processed using voxel-wise thresholds of *p* < 0.005 and *p* < 0.001, and an extent threshold of *k* > 20 voxels.

## Results

### Hormone assessment

**Figure 1:**
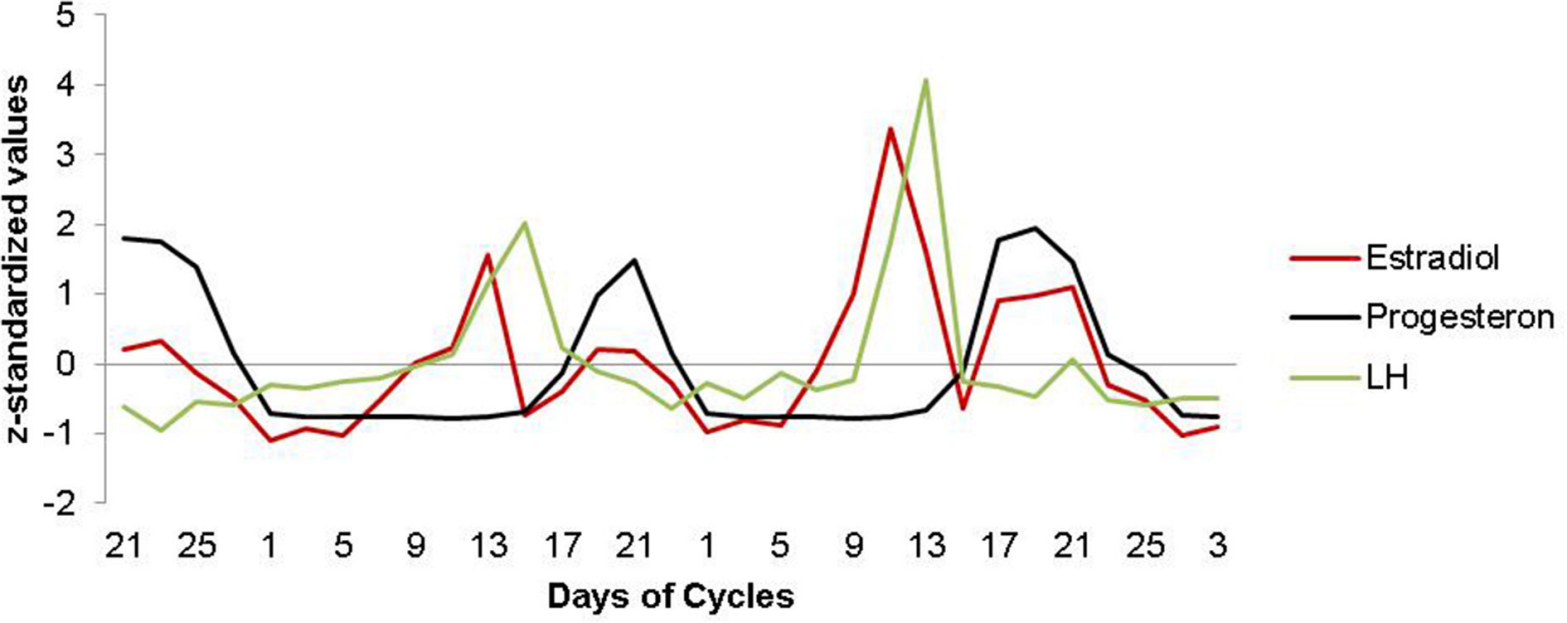
**Estradiol, progesterone and LH fluctuation across the menstrual cycle**. Characteristic patterns of serum estradiol (red line), progesterone (black line) and LH (green line) levels are displayed across two menstrual cycles. The depicted data represents days of cycle with corresponding z-standardized, single hormone values, matching the single scan time points in chronological order. As expected, estradiol shows a first prominent peak in the periovulatory phase, followed by a second peak in the late luteal phase. LH surges after the peak of estrogen, shortly before ovulation. Progesterone levels are low during the follicular phase and high during the luteal phase.

Hormone analyses confirmed characteristic patterns for estrogen and progesterone across all menstrual cycles assessed (for details, see Figure 1).

### EC mapping- whole brain analysis

**Figure 2:**
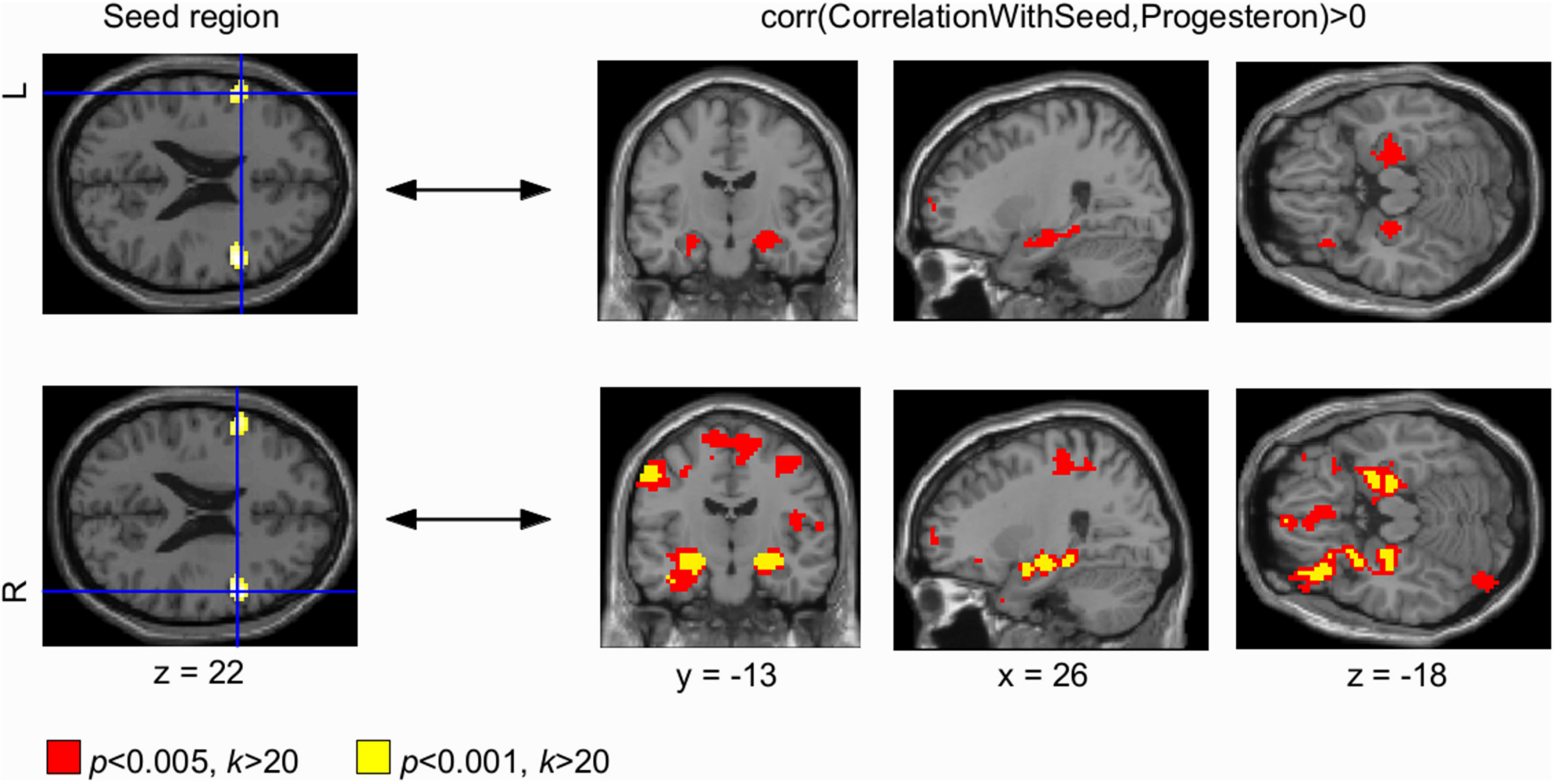
**Progesterone-modulated functional connectivity in bilateral dorsolateral prefrontal cortex and hippocampus**. The whole brain Eigenvector Centrality Mapping (EC) analysis reveals a significant correlation of progesterone with bilateral dorsolateral prefrontal cortex (DLPFC) (*p* < 0.001 unc.; **left panel**). In the seed based analysis, these progesterone-modulated intrinsic connectivity-changes in right and left DLPFC were found to connect with bilateral hippocampus **(right panel)**. For the left DLPFC (top panel), connectivity-changes to bilateral hippocampus were observed at a threshold of *p* < 0.005 (signal change depicted in red). The right DLPFC also showed progesterone-modulated connectivity with bilateral hippocampus modulated by progesterone at both thresholds: *p* < 0.001 (unc.; signal change depicted in yellow) and *p* < 0.005 (unc.; signal change depicted in red).

**Figure 3:**
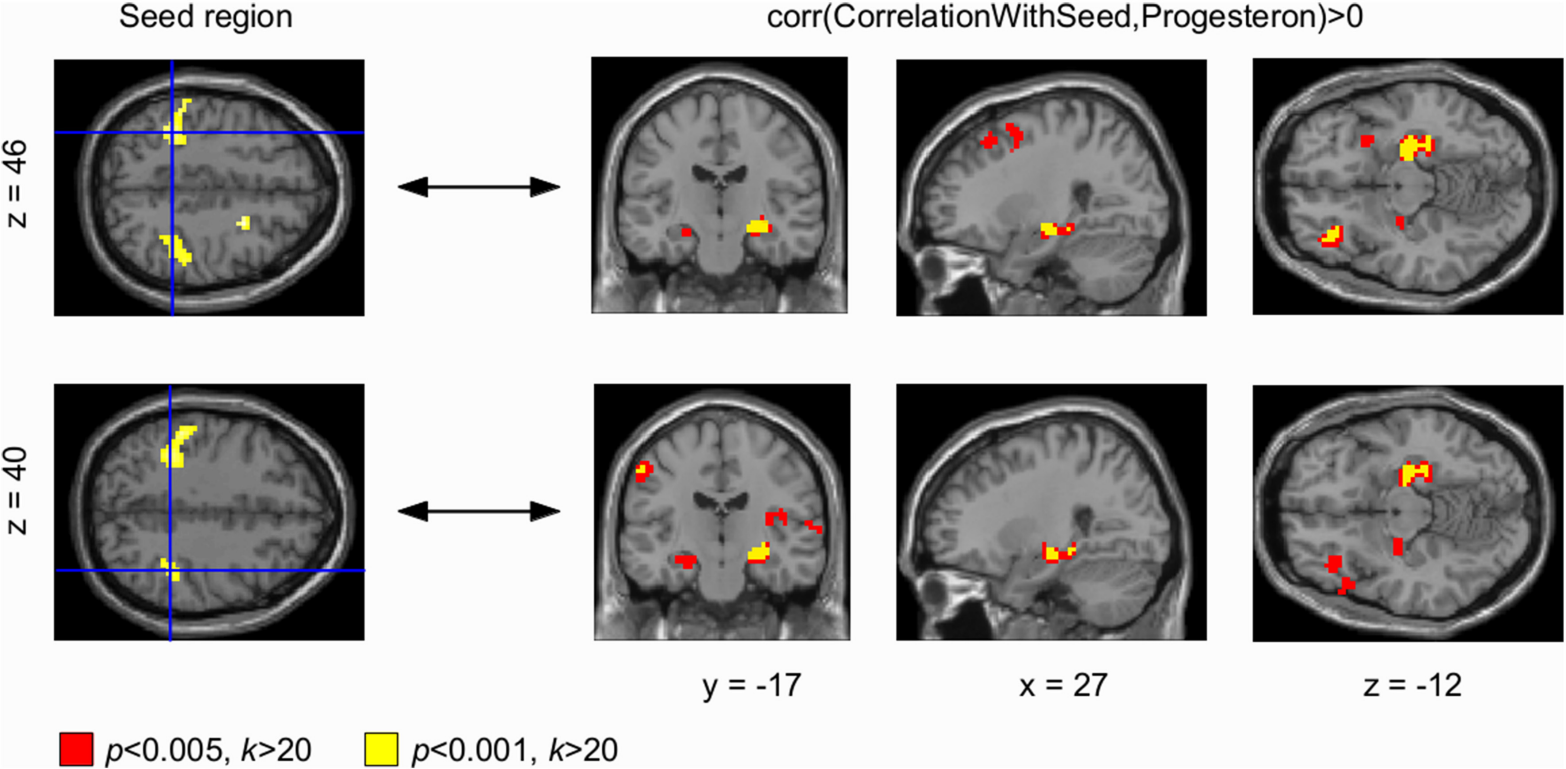
**Progesterone-modulated functional connectivity in bilateral sensorimotor cortex and hippocampus**. The whole brain Eigenvector Centrality Mapping (EC) analysis reveals a significant correlation of progesterone with bilateral sensorimotor cortex (*p* < 0.001 unc.; **left panel**). In the seed based analysis **(right panel)**, these progesterone-modulated intrinsic connectivity-changes in right and left sensorimotor cortex were found to connect with the left hippocampus (signal change depicted in yellow) at a threshold of *p* < 0.001 (unc.); for *p* < 0.005 (unc.) a bilateral effect with both hippocampi could be demonstrated (signal change depicted in red).

EC attributes a centrality value to each voxel in the brain such that a voxel receives a larger value if it is more strongly correlated with many other voxels, which are central within the network themselves. We found progesterone levels to modulate intrinsic functional connectivity across the entire brain, i.e., higher progesterone levels were associated with increased EC values in bilateral sensorimotor cortex and in the right dorsolateral prefrontal cortex (DLPFC) (Figures 2, 3, left panel; details of the analysis are provided in Table 1). We also found a cluster in the left DLPFC that did not survive the FWE correction. However, we report this cluster due to symmetry reasons. For whole brain analyses, cortisol was used as a covariate of no interest (for analysis-details, see Methods-Section). Neither positive nor negative correlations were found with estrogen.

**Table 1:** **Overview on detailed statistics (coordinates, cluster size, *p*-values) of results**.

**Set level**		**Cluster Level**				**Peak Level**							
***P***	***c***	***p*_FWEcorr_**	**α_FDRcorr_**	***k_E_***	***p*_uncorr_**	***p*_FWEcorr_**	**α_FDRcorr_**	***t***	***Z***	***p*_uncorr_**	**mm**	**mm**	**mm**
0.000	7	0.040	0.019	73	0.004	0.338	0.531	5.11	4.19	0.000	51	8	22
		0.548	0.156	23	0.078	0.538	0.531	4.80	4.00	0.000	24	5	46
		0.000	0.001	185	0.000	0.663	0.531	4.63	3.90	0.000	−51	−34	43
						0.708	0.531	4.57	3.86	0.000	−63	−31	31
						0.786	0.531	4.46	3.79	0.000	−36	−43	46
		0.191	0.058	43	0.021	0.752	0.531	4.51	3.82	0.000	−57	11	22
		0.101	0.037	55	0.010	0.986	0.738	3.93	3.44	0.000	57	−61	−5
		0.345	0.097	32	0.042	0.990	0.738	3.89	3.40	0.000	−21	−67	55
						0.993	0.738	3.85	3.38	0.000	−12	−73	58
		0.008	0.005	108	0.001	0.992	0.738	3.87	3.39	0.000	48	−34	46
						0.992	0.738	3.86	3.39	0.000	39	−43	40
						0.999	0.911	3.62	3.22	0.001	30	−46	52

*Table shows 3 local maxima more than 8.0 mm apart, p-values adjusted for search volume*.

*Height threshold: T = 3.34, p = 0.001 (1.000)*.

*Extent threshold: k = 20 voxels, p = 0.098 (0.630)*.

*Expected voxels per cluster, <k> = 7.511*.

*Expected number of clusters, ‹c› = 1.00*.

*FWEp: 6.123, FDRp: Inf, FWEc: 73, FDRc: 55*.

*Degrees of freedom = [1.0, 25.0]*.

*FWHM = 13.5 13.1 13.0 mm mm mm; 4.5 4.4 4.3 {voxels}*.

*Volume: 1672380 = 61940 voxels = 575.3 resels*.

*Voxel size: 3.0 3.0 3.0 mm mm mm; (resel = 85.56 voxels)*.

### Seed-based analyses

Seed based correlation analyses were used to find changes in BOLD signal correlations from centrality clusters to other areas across the brain. Our seed based correlation analyses revealed EC changes in right (and left) DLPFC (Figure 2, right panel) as well as bilateral sensorimotor cortex (Figure 3, right panel) to bilateral hippocampus to correlate with progesterone levels. We interpret these results as high progesterone to be paralleled with high connectivity between right DLPFC, bilateral sensorimotor cortex and hippocampus. For the left DLPFC (Figure 2, top panel), connectivity-changes to bilateral hippocampus were observed at a voxel-wise threshold of *p* < 0.005 (unc.), while the changes for the right DLPFC were significant at both voxel-wise thresholds *p* < 0.001 and *p* < 0.005 (unc.). In the seed based analysis of bilateral sensorimotor cortex (Figure 3, right panel), progesterone-modulated intrinsic connectivity-changes in the right and left sensorimotor cortex were found to connect with the left hippocampus at a voxel-wise threshold of *p* < 0.001 (unc.), and a bilateral effect with both hippocampi was demonstrated at *p* < 0.005 (unc.).

## Discussion

In the present study, we report the effects of the menstrual cycle on intrinsic connectivity in a single female subject, using a longitudinal design. In the initial whole-brain EC analysis, we find an FWE-corrected correlation of progesterone-levels with intrinsic connectivity changes in right DLPFC and bilateral sensorimotor cortex. Without FWE-correction, we also found a cluster in the left DLPFC that we also report due to symmetry aspects. Subsequent seed-based analyses demonstrate these progesterone-modulated intrinsic connectivity changes in right and left DLPFC connect to the bilateral hippocampus. We also observe a progesterone-modulated intrinsic connectivity change in bilateral sensorimotor cortex with the bilateral hippocampus.

A large body of evidence indicates a crucial role for the DLPFC in attention-demanding cognitive tasks involving domains of working memory and executive function (Curtis and D'Esposito, 2003), decision-making (Heekeren et al., 2006), reasoning (Goel and Dolan, 2004), problem solving (Ruh et al., 2012) and emotion-regulation (Levesque et al., 2003). The majority of treatment studies applying transcranial magnetic stimulation in unipolar depression target the DLPFC (Paus and Barrett, 2004; Padberg and George, 2009) for improved regulation of affective states, and enhanced cognitive control over stress and emotion responsiveness (Davidson et al., 2002). An essential feature of the DLPFC, which allows it to serve as a gate-keeper role across multiple neural networks, is its extensive interconnectedness with other brain regions, including thalamus, basal ganglia, orbitofrontal cortex, primary and secondary association areas of neocortex (Tekin and Cummings, 2002; Dosenbach et al., 2007) and hippocampus (Meyer-Lindenberg and Weinberger, 2006; Bilek et al., 2013). Dense pathways connect the DLPFC and the hippocampal formation (Goldman-Rakic et al., 1984), both directly and indirectly, and interactions between these regions are implicated in episodic memory (Simons and Spiers, 2003), the regulation of emotional–motivational states (Phillips et al., 2003), and experience-dependent plasticity (Bilek et al., 2013). Prefrontal (Brinton et al., 2008), and hippocampal (McEwen, 2002) regions are also exceptionally rich in the expression of sex steroid receptors. Evidence from animal work suggests sex hormones to be potent modulators of synaptic growth and density in regions of the prefrontal cortex and hippocampus (McEwen, 2002; Sanz et al., 2008; Galea et al., 2013). These findings are extended by human neuroimaging data that link changes in sex hormone levels with neural activity patterns in the above referenced regions during cognitive and affective tasks (Goldstein et al., 2005, 2010; Andreano and Cahill, 2010; Frank et al., 2010; Bayer et al., 2014). While systematic structural imaging studies exploring the effects of the menstrual cycle are still sparse, the limited evidence that has been collected comparing scans during the follicular and the luteal phase, or using between-group designs, support cycle-phase related changes in frontal cortical regions (Pletzer et al., 2010) and hippocampus (Protopopescu et al., 2008). Petersen et al. (2014) investigated intrinsic functional connectivity changes in users of oral contraceptives and compared the results to naturally cycling women (Petersen et al., 2014). The authors used ICA to evaluate the differences in resting state activity between these two groups. Of 20 compounds the analysis produced, 11 were discarded as likely artifactual findings and nine were retained (Petersen et al., 2014). These were identified amongst others as somatosensory, left frontoparietal, right frontoparietal, posterior default mode, anterior default mode and executive control networks. The anterior default mode and executive control network were selected as the networks of interest. Altered resting state dynamics could be shown in both networks, specifically the connectivity of the left angular gyrus, the left middle frontal gyrus and the anterior cingulate cortex were different between groups (Petersen et al., 2014). Luteal women showed reduced coherence between left angular gyrus and the rest of aDMN compared to follicular women. In the executive control network luteal women showed reduced coherence between right anterior cingulate and the rest of the network when compared to follicular women (Petersen et al., 2014). Users of oral contraception in the active pill phase showed reduced coherence between left anterior cingulate and the rest of the network as well as reduced coherence between the left middle frontal gyrus and the executive control network compared to naturally-cycling women in the follicular phase (Petersen et al., 2014). Our findings, particularly intrinsic connectivity changes in left DLPFC, partly correspond to these results, even though we found a specific modulation by progesterone and we did not analyze women on oral contraception as Petersen et al. found changes in connectivity in the left middle frontal gyrus, which is part of DLPFC, for women in the active pill phase compared to women in the follicular phase. Additional changes of intrinsic connectivity that we found, especially in sensorimotor cortex, may differ due to methodological reasons: limitation of Petersen et al. to the described two networks as well as the different analytical approach (ICA vs. EC). However, both studies show that there are changes of intrinsic connectivity during the menstrual cycle and provide evidence that endogenous hormones influence the baseline state of the brain (Petersen et al., 2014).

Our findings of progesterone-modulated intrinsic connectivity changes in the DLPFC and hippocampus are consistent with these converging lines of evidence from animal and human data that suggest the menstrual cycle influences structural and functional connectivity in sex-hormone receptor rich brain areas.

We also observed progesterone-modulated connectivity changes in the bilateral sensorimotor cortex in our whole brain analysis that revealed specific hippocampal connections in the subsequent seed-based analyses. These findings could be interpreted as menstrual cycle specific changes in hippocampal modulation of sensorimotor processes given that the hippocampus combines I) sensory afferents and synaptic mechanisms underlying rapid learning, as well as II) links to motivational, emotional, executive and sensorimotor functions (Bast, 2007). Specific to the menstrual cycle, it has been consistently reported that differences in pain perception are modulated by endogenous hormonal fluctuations (Riley et al., 1999), including areas within the sensorimotor cortex (Veldhuijzen et al., 2013). In women suffering from dysmenorrhea (severe menstrual pain), trait (Tu et al., 2010)- and state (Tu et al., 2013)-related gray matter changes were found in regions associated with pain modulation, pain transmission, and affective experience generation. Their findings include atrophic gray matter changes in the left secondary somatosensory cortex and hypertrophic changes in the primary somatosensory cortex (Tu et al., 2010, 2013), regions involved in sensory discrimination and interoception (Craig, 2002). Consistent with these results, our findings provide evidence for menstrual cycle modulated changes in functional connectivity in the somatosensory system.

The primary aim of the current study was to test the feasibility of extensive multiple longitudinal scanning sessions across the menstrual cycle for the resting state modality. A crucial limitation of the present longitudinal study was that it was conducted in a single subject. We acknowledge the limitations from this single-case design and the preliminary nature of our dataset. Our results have to be interpreted with caution and warrant replication in a larger sample. The current findings support the feasibility of combining rigorous menstrual monitoring and longitudinal, short-interval, intra-individual fMRI scanning protocols and thus, serve as a proof-of-principle study.

A portion of our findings, such as the EC changes in DLPFC, are consistent with the one study thus far (Petersen et al., 2014) that has reported functional connectivity changes across the menstrual cycle. However, Petersen et al did not show any significant association between either progesterone or estrogen levels with the connectivity changes observed in the aDMN or the ECN. Several methodological aspects could contribute to these controversial findings: Petersen et al. (2014) only assessed sex hormone levels at two different time-points during the menstrual cycle, once in the follicular phase and once in the luteal phase. As progesterone levels are only substantially elevated in the luteal phase, the variability in progesterone levels might not have been wide enough to capture any significant effects in a correlation analysis. While we have applied a whole brain approach, limiting the analysis to specific networks may have also affected their sex hormone correlation analyses.

We further acknowledge that the graph-based parameter EC measure is one that can be viewed as difficult to relate to brain function. Expert recommendations (Buckner et al., 2013; Sporns, 2014) have repeatedly stated that, while intrinsic functional connectivity provides a powerful and unique tool to provide insight into human brain organization, this method is also based on inherently ambiguous measures and limited by constraints from static anatomical connectivity and from poorly understood dynamic functional coupling changes. Thus, reports from resting-state studies are best interpreted in the context of external experiments. The converging evidence from animal and human literature (McEwen, 2002; Protopopescu et al., 2008; Galea et al., 2013), which suggest that the hippocampus is among the most susceptible of brain regions to subtle hormonal fluctuation, support our findings that link menstrual cycle phase to individual functional connectivity in this region.

We did not acquire perfusion data in our experiments, thus we are not able to directly test whether the EC changes we observed were influenced by blood flow. However, to date there is one study published that investigated perfusion changes in the female brain across the menstrual cycle: the authors report no cyclic variation in resting-perfusion using continuous arterial spin labeling (ASL) (Ances and Detre, 2003). Five female subjects were investigated at two times during their menstrual cycle: the initial scan was performed at the late luteal phase (1 or 2 days prior to the start of menses) and the second scan 4–5 days after completion of the menses (mid-follicular period). No significant differences in resting perfusion were observed for these two phases of the menstrual cycle. Liang et al. (2014) investigated cerebral blood flow (CBF) networks using ASL. The authors showed that highly connected brain regions overlap mostly with hub regions detected by BOLD fMRI studies. As expected, hub regions measured by ASL were characterized by shorter characteristic path length but higher vulnerability and eigencentrality. A positive nonlinear relationship of EC to regional CBF was shown. We cannot exclude that EC in general might be somewhat sensitive to perfusion changes. However, so far no evidence points to menstrual cycle specific perfusion changes and thus, we think it is unlikely that perfusion changes alone underlie the pattern of menstrual cycle specific EC-changes we report here.

In summary, our results suggest that the menstrual cycle substantially impacts intrinsic functional connectivity, particularly in brain areas associated with contextual memory-regulation, such as the hippocampus. We also observe progesterone-modulated changes in functional connectivity in bilateral DLPFC and sensorimotor cortex, regions that have been implicated in emotional regulation and pain modulation, domains that have previously been identified to be susceptible to menstrual cycle dependent rhythms. These results argue that the menstrual cycle is an important factor to consider when studying short-term functional plasticity in the human brain and highlight the importance of controlling for menstrual cycle phase in neuroimaging studies.

It is important to note that our data is of preliminary nature and warrants replication in a larger sample size. This longitudinal, single-subject study provides a first step toward the development of more individualized strategies of mapping important modulators of human brain states, such as the menstrual cycle. We applied rigorous menstrual monitoring and demonstrate the feasibility of longitudinal, short-interval, intra-individual fMRI scanning in combination with a simple analysis measure for whole brain intrinsic connectivity, which is what makes this pilot study stand out as a proof of principle. The current preliminary findings contribute to the development of more individualized mapping-strategies of the human brain by integrating potential mediators of brain states, such as menstrual cycle phase.

### Conflict of interest statement

The authors declare that the research was conducted in the absence of any commercial or financial relationships that could be construed as a potential conflict of interest.
