# Supplementary material for: Progesterone mediates brain functional connectivity changes during the menstrual cycle—a pilot resting state MRI study
Source: Front Neurosci. 2015 Feb 23;9:44. doi: 10.3389/fnins.2015.00044 (PMC4337344; doi:10.3389/fnins.2015.00044)

***Supplementary Material***

**Progesterone mediates brain functional connectivity changes in women during the menstrual cycle - A pilot resting state MRI study**

Katrin Arélin^a,b,c,§^, Karsten Mueller^a, §^, Claudia Barth^a^, Paraskevi Vivien Rekkas^d^, Jürgen Kratzsch^e^, Inga Burmann^a^, Arno Villringer^a,b,c,f,g^, Julia Sacher^a,b,*^

^a^ Max Planck Institute for Human Cognitive and Brain Sciences, Leipzig, Germany;

^b^ Clinic of Cognitive Neurology, University of Leipzig, Leipzig, Germany;

^c^ Leipzig Research Center for Civilization Diseases, University of Leipzig, Germany;

^d^ CAMH Research Imaging Centre and Campbell Family Mental Health Research Institute at the Centre for Addiction and Mental Health and the Department of Psychiatry, University of Toronto

^e^ Institute for Laboratory Medicine, Clinical Chemistry and Molecular Diagnostics, University Hospital Leipzig, Leipzig, Germany

^f^ Integrated Research and Treatment Center Adiposity Diseases, University of Leipzig, Germany;

^g^ Berlin School of Mind and Brain, Mind and Brain Institute, Berlin, Germany;

^§^ These authors contributed equally to this work

^*^ corresponding author

## 1. Supplementary Figures

## 1.1. Figure A

Figure A shows the general maps of EC averages.

Our results are in agreement with other investigations, such as the general maps of EC published by Lohmann et al. (2010). We find similar patterns of increased EC in thalamus, motor areas, and wide cerebellar regions when averaging all EC maps over time.


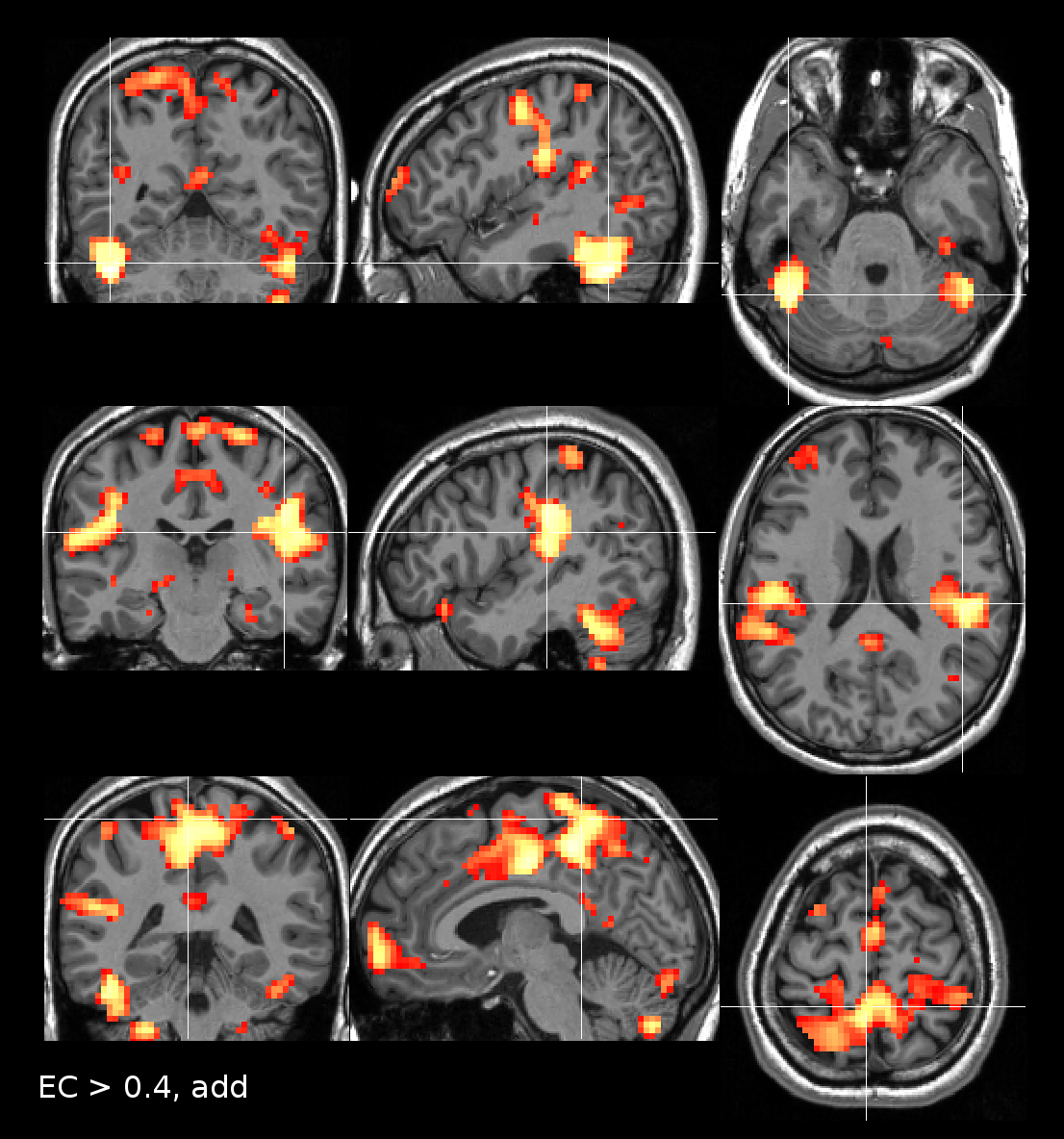


## 1.2. Figure B

## Figure B shows the EC maps for both scanning sessions.

## The general maps of EC averages were highly consistent between both scanning sessions. Both averages show an increased EC signal in motor and cerebellar regions but also in the primary auditory cortex and the left DLPFC.


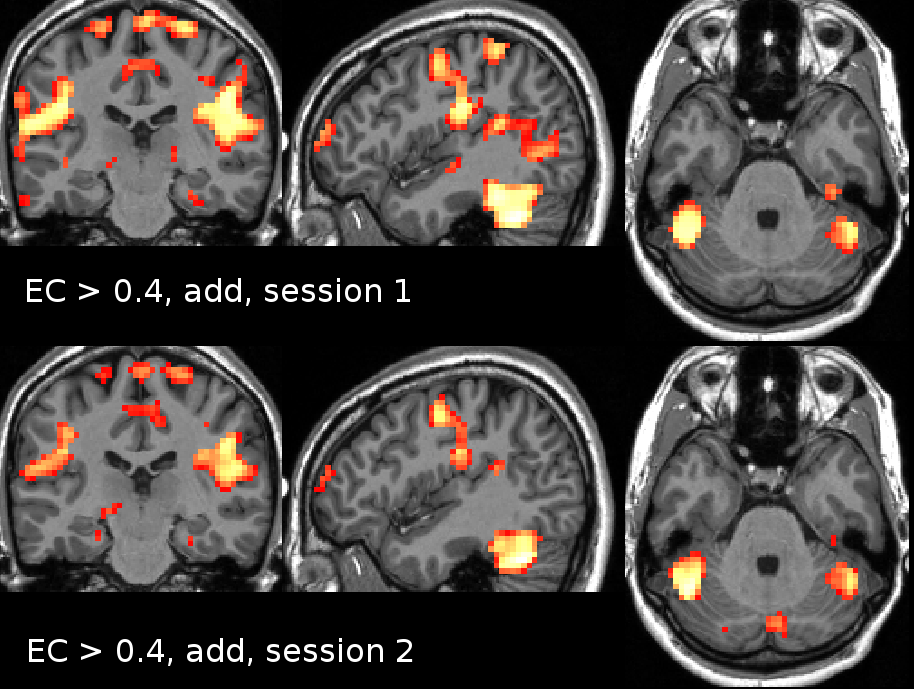


## 1.3. Figure C

Figure C shows the consistent relationship between progesterone and EC over sessions.

We performed a correlation analysis between progesterone and EC for each scanning session separately and found a consistent significant correlation between progesterone and EC in the DLPFC and somatosensory cortex (*p*<0.01, see overlapping orange regions).


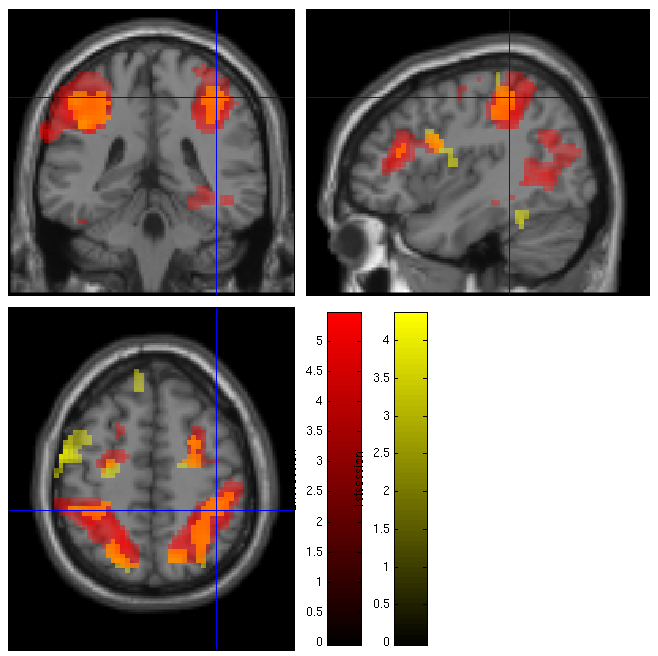

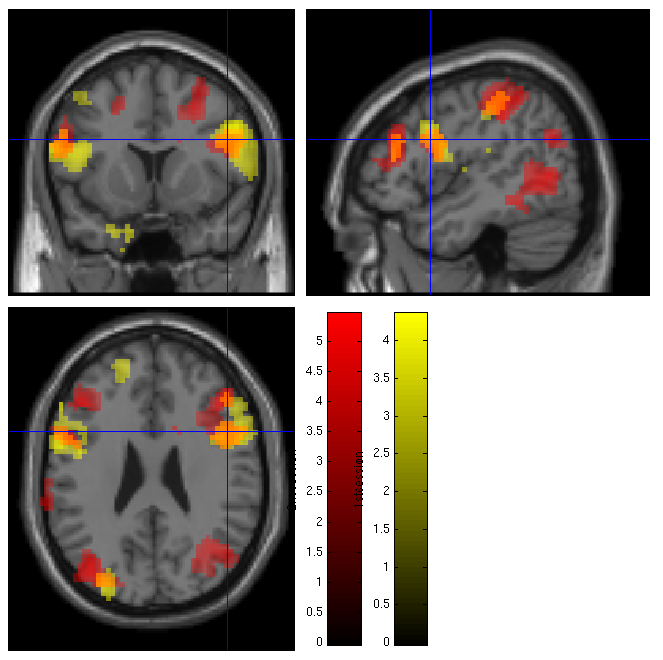

Supplement: Supplementary file 1 [file DataSheet1.DOCX]
